# Some of the Pathological Conditions of the Oral Cavity

**Published:** 1885-10

**Authors:** F. D. Nelles


### SOME OF THE PATHOLOGICAL CONDITIONS OF THE ORAL CAVITY.

BY F. D. NELLES, D. D. S.

READ BEFORE THE FIFTH DISTRICT DENTAL SOCIETY OF THE STATE OF NEW YORK.

In fulfillment of the conditions of the requirements for such a paper as you have asked of me, nothing would have been easier than to have turned to some good pathological treatise and given a classified list of the various diseases having their seat in the oral cavity, of which all of us know more or less, theoretically, but which no two would treat exactly the same when the practical case is presented.

To give this assertion more the semblance of a fact, allow me to cite the following examples : A regular patient came to the office one day, asking the cause of a certain prominence located in the maxillary process of the superior jaw. There was no aching, no pain, no redness of the gums, and no inflammatory action apparent. Neither was there any soreness of the incisors over which the fullness was located. Now, what was the difficulty ? Let us examine the case a little further. The age of the patient was fifty years or more; the teeth were abraded. Did that mean anything ? Might there not be a putrescent pulp in one of the teeth ? A brother practitioner happening to be present at the time, and being desirous of ascertaining whether two dentists could agree in tracing effect to cause, he was invited to assist in clearing up the mystery, for as such it must be considered until all doubt was set at rest. That it was something abnormal we were well agreed, but differed as to the mode of treatment. He recommended painting over with a solution of iodine; but why not as well paint the integument of the face, including the alæ of the nose and a portion of the lip, where the fullness was quite visible? The advantage, however, was mine, as the patient had come to me. I announced that we would treat the case heroically, and he, with some astonishment, saw me select a bur of proper size and drill through to the pulp-chamber. A free discharge of pus quickly followed, and the treatment thereafter was very easy. That was more than nine years ago, and the tooth now, abraded to the very gums, is still doing good service.

It might be well to observe that a collection of pus, under such circumstances, when unattended with redness, tenderness, and devoid of all inflammatory signs, is said to be either a chronic or cold abscess, yielding a pus markedly different from that which is designated as laudable, it being much thinner and more sanious.

The other case to which I desire to call your attention, was that of a girl about twelve years of age, who complained of two very sore teeth, the inferior central incisors. The appearance of the countenance and eyes was such as invariably accompanies affections of anginous origin. The teeth were not only sore, but loose, and raised from their sockets. For comfort, the jaws were kept well asunder, causing the saliva to run unrestrained from her mouth. The gums were not red nor swollen, but unnaturally light in color. She had received no blow or fall, no injury of a mechanical nature. Now, what could have been the cause of the trouble?

Let us look into the case a little further. Just behind the front teeth lie two little glands—the sublinguals. They were found to be enlarged beyond the normal size, but were free from any inflammatory signs. Such being the case, how could one conscientiously decide that they were the cause of the trouble. A prescription, based upon this supposition, was, however, made: ℞ Ammonia Chloridi, drachms two, water sufficient to make four ounces, to be held in the mouth in teaspoonful quantities several minutes before spitting out, fifteen or twenty times a day until all pain was gone, then to come again.

In about four weeks two little girls came to the office, one to have a tooth extracted. When she had been disposed of I looked at her companion and recognized in her the girl of the sore teeth. When asked why she had not come back the following day as requested, she replied, "Oh, the teeth got well right away," and upon looking I found she had spoken truthfully, as there was then nothing remaining to indicate that the teeth had ever been affected.

I have arrived at that point now, where I hope we shall begin to understand that in treating diseases of the mouth successfully, much of our skill lies in a clear-sighted diagnosis. The oral cavity has been the *open sesame* to most physicians of the past, and will continue to be the object of examination just so long as it supplies a talismanic guide to distinguish the difference between certain kinds of diseases preying upon the human system. If diseases of the general system can be the better established by conditional aspects of the mucus membrane of the mouth for physical practice, why not for dental practice as well? Rheumatism, when it has become chronic, will affect the teeth and gums inversely, as other organs of the body. With this disease we may look for inordinately pale gums and resorption of the alveolar process, deposition of tartar, reaching from the necks of the teeth to the receded gums, an eighth of an inch, more or less. Both upper and under jaws will be affected about alike, and the recession of the gums around the incisors and bicuspids pretty even, showing most on the labial and buccal surfaces. The tartar will be found light in shade, tenacious, and intruding underneath the free edge of the gums. Creamy deposits of food and fatty matters will be found adhering to the tartar. Touched with litmus paper, it will always give an acid reaction. The teeth will be more or less decayed on the labial and buccal surfaces. The treatment will consist in removing the tartar, filling the cavities, and giving alkaline washes for the mouth and teeth. Sulphite of soda, one-half drachm to about eight ounces of water, makes an excellent detergent. One ounce of this solution poured in a cup in which the brush is repeatedly dipped, holding some of it in the mouth while brushing, will thoroughly cleanse the teeth and mouth. It is a little unfortunate, perhaps, that in the treatment of such diseases we have so small a field as the mouth affords to labor in. Had we the power accorded to the family physician, in treating diseases of a general origin that produce such devastating effects upon the teeth, we might in many cases accomplish more pleasing results.

It will never do for us, any more than for physicians, to ignore the fact that the mucous membrane, all of that portion of it which lines the mouth, invites our especial attention. The teeth, although dense and flint-like in structure, are of dermic origin, and derive their nutrition wholly from this source. Is it not natural, therefore, to reason that any disease having its seat in this membrane in more ways than one will affect the teeth? If acute adenomata of the sublingual glands will do this, may we not assume that other glands, if even more remote, may produce similar disturbances? When examining pathological works touching upon this subject, most of them will be found more or less explicit in tracing affections of the mucous membrane from structure to structure, tissue to tissue, until all abnormal changes are traced, save those affecting the teeth. For further knowledge we must turn to dental pathology, where we find much about teeth, and little, too little, about the soft tissues. It is true we now and then get an essay from some bright mind, theoretically advocating that all, or nearly all, diseases of the mouth spring from carious teeth, but another soon comes forward with the argument that all, or nearly all, such diseases arise from calcareous deposits upon the teeth. Still another will proclaim it is neither the one nor the other, but that the material which is put in carious teeth as fillings is the cause of all, or nearly all, the diseases incidental to the mouth. So, when we come to prescribe for diseases of the oral cavity, if we trust entirely to the books, we shall continue in darkness.

The gums may be atrophied or hypertrophied, it matters but little which. The inflammatory redness accompanying gingivitis will never be wanting. If, however, an atrophied condition prevails, the treatment will be found comparatively easy, as nothing much will be required but removal of the tartar, found almost invariably free from and above the gums. But this state of things is entirely different when the gums are in a hypertrophied condition. Then the free edge of the gums will be much thickened and operculous. The fibrous attachments running from the tooth membrane to the gums will seem absorbed. Each and every tooth will seem placed in the gums mechanically, with a little dirt of a dark or lightish hue thrown around them, as if in hope that they may some day take root and grow. But a more careful examination will show this to be tartar. The treatment will consist in removing the tartar with alkaline washes, sulphite of soda, sulphate of potash or borax, given in solution, one to two drachms in four to eight ounces of water. Give them singly. Sometimes the potash will do more good than the soda.

A solution of tannic acid with alcohol will sometimes do better in such cases than alkaline washes. One objection to it is, that it will stain the exposed cementum at the necks of the teeth, if kept up too long. Two to three grains of tannin to one ounce of undiluted alcohol will be sufficiently strong. A better formula, however, would be the following:

Tannic Acid, grs. iii.
Alcohol absolute, drachms iii.
Water distilled, do. v.

Add the tannin to the water. Shake, and then add the alcohol. It may be flavored with two to five drops of oil of wintergreen. This must be dropped into the alcohol previous to adding it to the water and tannin. The manner of using this wash has much to do with its ultimate success. The instructions should be to brush the teeth as usual, with water, rinse out the mouth, and wash out the brush; now pour on the brush as much of the solution as it will hold without wasting, and go over the teeth and gums with the brush carefully, as if they had not been previously brushed.

